# Root-associated microbiota drive phytoremediation strategies to lead of *Sonchus Asper* (L.) Hill as revealed by intercropping-induced modifications of the rhizosphere microbiome

**DOI:** 10.1007/s11356-021-17353-1

**Published:** 2021-11-19

**Authors:** Xinyue Mei, Ying Wang, Zuran Li, Marie Larousse, Arthur Pere, Martine da Rocha, Fangdong Zhan, Yongmei He, Linlong Pu, Franck Panabières, Yanqun Zu

**Affiliations:** 1 College of Resources and Environment, Yunnan Agricultural University, Kunming, 650201 China; 2 State Key Laboratory for Conservation and Utilization of Bio-Resources in Yunnan, Yunnan Agricultural University, Kunming, 650201 China; 3 College of Landscape and Horticulture, Yunnan Agricultural University, Kunming, 650201 China; 4 Université Côte d’Azur, INRAE, CNRS, ISA, 06903 Sophia Antipolis, France

**Keywords:** Hyperaccumulator, Intercropping, Microbiome, *RNA-Seq*, *Sonchus asper*, *Zea mays*

## Abstract

Intercropping or assistant endophytes promote phytoremediation capacities of hyperaccumulators and enhance their tolerance to heavy metal (HM) stress. Findings from a previous study showed that intercropping the hyperaccumulator *Sonchus asper* (L.) Hill grown in HM-contaminated soils with maize improved the remediating properties and indicated an excluder-to-hyperaccumulator switched mode of action towards lead. In the current study, RNA-Seq analysis was conducted on *Sonchus* roots grown under intercropping or monoculture systems to explore the molecular events underlying this shift in lead sequestering strategy. The findings showed that intercropping only slightly affects *S. asper* transcriptome but significantly affects expression of root-associated microbial genomes. Further, intercropping triggers significant reshaping of endophytic communities associated with a ‘root-to-shoot’ transition of lead sequestration and improved phytoremediation capacities of *S. asper*. These findings indicate that accumulator activities of a weed are partially attributed to the root-associated microbiota, and a complex network of plant–microbe-plant interactions shapes the phytoremediation potential of *S. asper*. Analysis showed that intercropping may significantly change the structure of root-associated communities resulting in novel remediation properties, thus providing a basis for improving phytoremediation practices to restore contaminated soils.

**Supplementary Information:**

The online version contains supplementary material available at 10.1007/s11356-021-17353-1.

## Introduction

Heavy metal pollution of agricultural soils constitutes one of the most significant environmental pollutions worldwide (Huang et al. 2018). China has large-scale mineral resources and is among the highest global producers and consumers of metals (Li et al. 2014). Notably, long-term mining and smelting activities pose a high risk of toxic metal pollution, mainly in the southeast areas of the country (Yang et al. 2018). For instance, Huize mining area is a representative area of large-scale lead–zinc deposits in Yunnan province, China (Huang et al., 2003). Metal pollution causes deleterious damage on soil microbial communities and plant growth. Non-essential metals such as lead (Pb) have noxious effects, easily penetrate plant tissues and affect growth and general metabolism processes such as nutrient uptake and photosynthesis (Gopal and Rizvi 2008; Alves et al. 2014). Therefore, studies should explore effective methods for elimination of heavy metals from contaminated soils.

Phytoremediation is a low-cost, environment friendly, sustainable and effective method for restoring soil integrity. The approach comprises use of plants that extract and sequester metals from soils without significant deleterious effects (Salt et al., 1998; Mahar et al. 2016). Some herbaceous plants such as *Arabis alpina Var. parviflora* Franch, *Cynodon dactylo*n L. Pers, *Malva verticillata* L. and *Chenopodium ambrosioides* L. grow naturally around lead–zinc mine tailing areas and accumulate different heavy metals (Li et al. 2019; Zhan et al. 2019; Mayerová et al. 2017; Zhang et al. 2012). *Sonchus asper* (L.) Hill is an annual herbaceous dicot which probably originates from the Mediterranean Basin (Hutchinson et al. 1984). *S. asper* can accumulate lead and cadmium and is present in Huize lead–zinc mining areas (Zu et al. 2005). Therefore, these plants can be used as phytoremediators. They can act as excluders by maintaining a relatively low concentration of metals in above-ground tissues compared with the level in roots (Baker 1981) or hyperaccumulators with a strongly enhanced rate of metal uptake and accumulation in leaves without any toxicity effects (Rascio and Navari-Izzo 2011; Corso et al. 2018; Wang et al. 2020). Hyperaccumulators have been widely explored for phytoremediation purposes (Verbruggen et al. 2009; Li et al. 2018). However, hyperaccumulators have limitations such as slow growth rate, low biomass production and low restoration rate (Maestri et al. 2010). Therefore, studies should explore strategies for combining hyperaccumulators with other measures to improve efficiency of heavy metal remediation (Chen et al. 2019).

Previous studies have explored the ability of hyperaccumulators to recruit beneficial microbes to enhance growth and tolerate environmental stress, including heavy metal stress (Miransari 2011; Hou et al. 2018). Therefore, combining use of hyperaccumulators and beneficial microorganisms to promote phytoremediation processes is a promising alternative strategy for improving phytoremediation (Gupta and Joia 2016). This approach makes use of the association among assistant endophytes, rhizospheric bacteria and/or fungi and plants to eliminate metals from contaminated soils by enhancing plant biomass production and facilitating phytoextraction or reducing phytostabilization (Glick 2010; Ma et al. 2016; Sharma et al. 2019). For instance, *Arabis alpina* hosts highly complex fungal and bacterial communities that significantly promote heavy metal tolerance (Sharma et al. 2019; Sun et al. 2019). Moreover, dark septate endophytes (DSEs) which is a diverse group of ascomycetes (Jumpponen and Trappe 1998) and arbuscular mycorrhizal fungi (AMFs) which belong to the phylum Glomeromycota among Mucoromycetes (Brundrett and Tedersoo 2018) establish endophytic relationships with their hosts resulting in improved metal sequestering properties of the plant (Pawlowska et al. 2000; Torrecillas et al. 2013). These organisms directly participate in soil remediation process owing to their ability to degrade organic contaminants thus decreasing metal toxicity, in addition to playing a role in promoting plant growth, and metal accumulation and translocation (Ma et al. 2016; Zhan et al. 2019). However, high levels of metal in soils may affect the structure and dynamics of rhizosphere microbiome.

Intercropping is a widespread cultural practice in Asian countries, mainly in China (Knoerzer et al. 2010). This agricultural practice comprises growing two species together, with an expected benefit on both partners in terms of improved nutrition and yield, as well as increased resistance to pathogens (Gaba et al. 2015; Zhu and Morel, 2019). In addition, intercropping can be performed between hyperaccumulators and other plants for remediation of heavy metal-contaminated soils (Wu et al., 2007; Hussein et al., 2019). Our group conducted a preliminary study and showed that phytoremediation capacities of *S. asper* were improved when the plants were grown under intercropping system with maize (*Zea mays*), a food crop widely grown in Yunnan Province (Pu et al., 2018). Other studies report that Sonchus roots are commonly infected by both DSEs and AMFs in natural environments (Massenssini et al. 2014) and that soilborne microbial communities can be altered by intercropping (Zhu and Morel 2019). These findings suggested that *S. asper*, as an excluder, developed efficient ways to retain, sequester and possibly detoxify the metal within roots, probably through the help of its associated microbiota. Therefore, we intended to explore the effect of intercropping *S. asper* with maize on the global remediation properties, with a focus on changes in the microbiota composition and the global hyperaccumulator properties of *S. asper*.

Therefore, the current study evaluated the effect of intercropping on the global transcriptome of Sonchus root system through RNA-Seq analysis. We show here that intercropping only slightly affects Sonchus transcriptome but significantly affects composition and expression of associated microbial genomes. Moreover, intercropping causes a switch from an excluder to accumulator behavior towards lead, indicating the possible effect of the endophytic community of *S. asper* to the remediation effect of lead. These findings provide a basis for studying plant-plant interactions to further explore the effect of biotic associations on phytoremediation.

## Materials and methods

### Soil and plant materials

Lead-contaminated soil was collected from a farmland around the lead–zinc mining area in Huize, China. The mining area is located in the north-east of Yunnan Province, China. Soils were sampled from the surface, with a maximum depth of approximately 20 cm. The soil comprised 1540 mg kg^−1^ total nitrogen, 59.98 mg kg^−1^ alkali nitrogen, 28.2 mg kg^−1^ available phosphorus, 122 mg kg^−1^ available potassium, 28.2 cmol kg^−1^ Cation Exchange Capacity (CEC), 16,100 mg kg^−1^ organic matter, 600 mg kg^−1^ lead, and the pH was 5.5. Sonchus asper seeds were harvested from the Huize Lead–Zinc Mine at maturity stage. Seeds from the low-grade Pb-accumulation maize (*Z. mays*) cultivar Huidan No. 4 (Shen et al., 2018) were purchased from Kunming Xiaobanqiao Seed Station. Seed surfaces were sterilized with 6% H_2_O_2_ for 10 min followed by four successive washes with sterile distilled water. Germination of seeds was performed on moist filter paper before transplantation.

### Pot experiments in greenhouse and analysis of heavy metal levels

The experiments were conducted partially following methods described previously (Pu et al., 2018). Greenhouse experiments comprised two planting patterns (Supplementary Figure S1). The monoculture of *S. asper* (MS) comprised planting of three plants in each pot, and a maize plant was placed between two *S. asper* plants in the intercropping (MS) pattern. A 10-cm intra-row spacing was allowed between every two plants in the planting systems. Each pot (40-cm diameter, 15-cm depth) comprised 6-kg dry soil. Three biological replicates were used for each treatment. Pots were placed in a greenhouse with frequent irrigation to avoid drought stress and maintained under a 12 h/12 h day/night photoperiod and a temperature ≤ 30 °C. Plants from each replicate were harvested after 60 days. *S. asper* samples were rinsed with tap water, then further washed with deionized water to eliminate soil contamination. Samples were wrapped then rapidly air-dried at 105 °C for 30 min, then dried separately in an oven at 75 °C for 72 h, then the dry weight (DW) was determined. Collected samples were assigned into two subsamples as follows: half of Sonchus and maize root samples were washed with distilled water, frozen in liquid nitrogen, and stored at − 80 °C fur subsequent RNA isolation. The other root and shoot samples were used for determination of biomass, heavy metal concentration and rhizosphere soil metal content.

Concentrations of Pb in plants were determined using 0.5 g of roots and shoots after digested with HNO_3_-HClO_4_ (3/1, v/v) as described previously (Tanvir et al. 2014). Transport characteristics of Pb were expressed by translocation factor (Zu et al., 2005). Differences between Pb concentration among shoots and differences among different roots were determined by one-way analysis of variance (ANOVA) followed by Fisher’s least significant difference (LSD) test (*p* < 0.05). Statistical analysis was performed using PASW Statistics 18 (SPSS Inc., Chicago, IL).

### RNA isolation and RNA-Seq

Total RNA of Sonchus roots grown under monoculture (MS) or intercropping (IS) conditions was isolated using Trizol reagent (Promega, USA) following the manufacturer’s instructions. RNA samples were treated with RNase-free DNase I (Takara Bio, Japan) for 30 min at 37 °C to remove residual DNA. RNA concentration was determined using a 2100 Bioanalyzer at 260 nm and 280 nm, and samples exhibiting a 260 nm/280 nm ratio between 1.8 and 2.0 were used in subsequent analyses. Poly (A) mRNA was isolated using oligo-dT beads (Qiagen). mRNA was fragmented (200 nucleotides) and reverse transcribed using random hexamers, followed by second-strand synthesis. cDNA fragments were purified by agarose gel electrophoresis after purification, end repair, poly (A) tailing and adapter ligation. cDNA was extracted from gels then enriched by PCR to construct the final cDNA library. Two cDNA libraries from each condition were sequenced on an Illumina sequencing platform (Illumina HiSeq™ 2500) using the single-end paired-end technology in a single run. Original images were processed to sequences, then base-calling and quality value calculation were performed using the Illumina GA Pipeline (version 1.6) and 100-bp paired-end reads were obtained (Li et al., 2013).

### Bioinformatics analyses and sequence manipulation

Illumina reads from the four libraries were mixed and assembled using Trinity (Haas et al. 2013), after removal of low-quality sequences, reads harboring more than 5% N (unknown) bases and reads containing untrimmed adaptors to generate the unigene dataset. Completeness of the transcriptome assemblies was assessed using BUSCO metric (Simão et al. 2015) with eukaryotic gene set and Viridiplantae gene set as references. Unigenes were assigned to a given organism using a best hit BLAST approach against GenBank using the following criteria: query sequences matching nr hits with ≥ 70% identity on ≥ 50% of their length and an e value ≤ e-04 implied that they belonged to a given organism. Sequences were further classified in the tree of life using a non-hierarchical classification system reported previously (Adl et al., 2019). Functional annotation included Blastn searches, as well as translation of unigenes and subsequent searches in NR and KOG/COG using default parameters. Transposable Elements (TEs) were identified by Blastn searches against the RepBase database using the Censor tool (Bao et al., 2015). Functional domains were predicted by alignment of ORGs of the unigenes against PFAM (pfam.xfam.org) and PROSITE (https://prosite.expasy.org) databases. Presence of potential signal peptides was determined using SignalP v5 (Almagro Armenteros et al., 2019). Prediction of transmembrane helices was performed using the HMMTOP server (www.enzym.hu). Nuclear export signals were retrieved from the NetNES server (cbs.dtu.dk). Fungal effectors were predicted using Effector2P tool (Sperschneider et al., 2016).

### Determination of differentially expressed genes (DEGs)

Sample reliability analysis was conducted using Pearson correlation analysis. Expression levels of individual libraries were determined using the RPKM (Reads per kilobase transcriptome per million mapped reads) method (Mortazavi et al., 2008). Differentially expressed genes (DEGs) between MS and IS libraries were defined by false discovery rate (FDR), with *P* values ≤ 0.001 and a log_2_| fold change |≥ 2.

### Validation of expression profiles of DEGs by qRT-PCR

qRT-PCR was performed on a set of 14 genes that were randomly selected among the DEGs to verify the expression profiles revealed by RNA-Seq. cDNAs were reverse transcribed from the RNA isolated by Trizol reagent (Invitrogen) then used for the qRT-PCR validation. Samples were ground in liquid nitrogen, and total RNA was isolated using Trizol reagent. Integrity of total RNA was determined using agarose gels, and its quantity and purity were determined spectrophotometrically. A total of 50 ng to 2 µg of RNA was used as a template for reverse transcription reaction in a 20-µL reaction volume using the FastQuant RT Kit (TIANGEN; www.tiangen.com). Real-time qRT-PCR was carried out using qPCR Mastermix for TIANGEN. Reactions were run, and quantification was performed using the ABI StepOnePlus Real-Time PCR Detection System (Applied Biosystems; www.thermofisher.com). PCR for each biological replicate was performed using three technical replicates. Two-microliter cDNA and 0.4-µm primers (Supplementary Table S3) were used for each reaction. The initial denaturing time was 2 min, followed by 45 PCR cycles at 95 °C for 5 s (denaturation), 60 °C for 15 s (annealing) and 72 °C for 20 s (elongation). Specificity of the amplification was confirmed by a single peak in a dissociation curve at the end of the PCR procedure. The stability of the reference gene across samples for each experiment was determined using the Normfinder software (Andersen et al., 2004). Data were analyzed using RqPCRBase R package for analysis of quantitative real-time PCR data (Hilliou and Tran 2013). Differences were quantified in target gene expression based on a standard control condition (MI). mRNA levels were normalized against the constitutive expressed actin gene (Paolinelli-Alfonso et al., 2016). Amplifications for each biological replicate were performed in three technical replicates. Quantitative PCR values were transformed by log2 Ratio (IS value/MS value) for comparison of gene expression variation between RNA-Seq and qRT-QPCR data.

## Results

### Intercropping improved phytoremediation transport characteristics of Sonchus asper towards lead

Intercropping with maize caused a significant increase in biomass of Sonchus roots and shoots (23.25% and 42.85%, respectively) compared with the monoculture system (Supplementary Table S1). In addition, Pb content significantly increased in Sonchus plants compared with the monoculture system. Further analysis showed changes in *S. asper* characteristics towards Pb, with a significant increase in lead content in aerial parts of the plants and decrease in Pb content in root tissues (Supplementary Table S2). Moreover, the translocation factor increased from 0.835 to 1.795, resulting in a switch from excluder to hyperaccumulator behavior towards lead (Supplementary Table S2). Determination of Pb content in roots and shoots of maize showed a significant decrease in the content in shoots and roots, compared with the values obtained under monoculture conditions (Supplementary Figure S2). These findings indicate the beneficial effect of the intercropping strategy for phytoremediation consistent with findings reported previously (Pu et al. 2018). In addition, the findings indicate that intercropping reduced the level of contamination of maize by lead (Supplementary Figure S2).

### RNA-Seq analysis reveals the complexity of the Sonchus root-associated microbiota

The transcriptional profile of the roots of Sonchus grown under monoculture and intercropping conditions was determined to explore the possible causes of the observed excluder-to-hyperaccumulator switch in Sonchus after intercropping. This analysis was performed to determine the transcriptional activity of the biotic community directly associated with root tissues. Previous studies reported that the root microbiota are implicated in the bioremediation properties of the plant, and that possible changes in composition and/or activity of the biotic community upon intercropping modulate the hyperaccumulator behavior of Sonchus (Hao et al., 2021; Sharma et al., 2021; Wang et al., 2021). The current study sought whether intercropping with maize would induce a root-to-shoot translocation of lead through activation of transporters, and/or inhibition of the mechanisms implicated in accumulation of the metal in roots in monoculture conditions.

Several libraries were constructed from mRNA of Sonchus roots grown either under monoculture (MS) or under intercropping (IS) systems, and RNA-Seq data were generated. Sequencing yielded ~ 40 million, 125-bp long reads, and 98.45% high quality clean reads were obtained for each library. Reads were pooled for de novo assembly using Trinity because *S. asper* genome sequence was not available. The analysis resulted in 65,357 contigs with an average length of 714 bp and N50 length of 1095 bp. We identified 297 of the 303 evolutionary conserved BUSCO eukaryotic genes, with only 1.98% of BUSCO genes missing from the assembly (Supplementary Table S4). The overall completeness score was 92.32% using the 430-gene plant dataset as reference (Supplementary Table S4). So, sequence coverage was sufficient for further analyses. The quality of the assembly was explored by determining the actual occurrence of unigenes in individual libraries, and 8595 sequences that were not represented by at least one read in at least one library were discarded. Therefore, a 56,762-unigene dataset was used for subsequent analysis. A best hit Blastn analysis was performed against nr hosted at GenBank to identify the origin of the mRNAs corresponding to the unigenes. A total of 20,339 unigenes could not be accurately assigned to a given organism and were considered of ‘unknown origin’ (Supplementary Figure S3). Partial matches to GenBank entries and weak homologies can produce false positive results; thus, additional parameters were added for analysis, such as the percentage of identity, the length of the unigene matching an entry, the e value, as well as a minimal amount of reads in at least one library to verify that the unigene is actually expressed and does not correspond to a contamination (see Materials and methods). Retained sequences (16,878 unigenes, Supplementary Figure S3) were mainly of eukaryotic origin, while 22 unigenes highly matched viral sequences, and 46 unigenes were assigned to bacterial origin (Supplementary Table S5). Yet, analysis did not indicate whether these prokaryotic sequences were derived from root-associated bacteria, if they were hosted by organellar genomes of eukaryotic hosts, or if they have been acquired laterally. Further analysis of probable eukaryotic sequences showed 91 fungal sequences and 200 unigenes of animal origin that had better analogs mainly among nematodes, chordates and arthropods. So, although the sequences likely derived from plant origin constituted significantly higher proportion of the dataset (98.02%, Supplementary Table S5), the Sonchus root-derived transcripts may have diverse origins. Further analysis was thus conducted to compare the biotic, transcriptionally active assemblages in both monoculture and intercropping conditions.

### Intercropping significantly affects composition of Sonchus associated biotic community

**Fig. 1:**
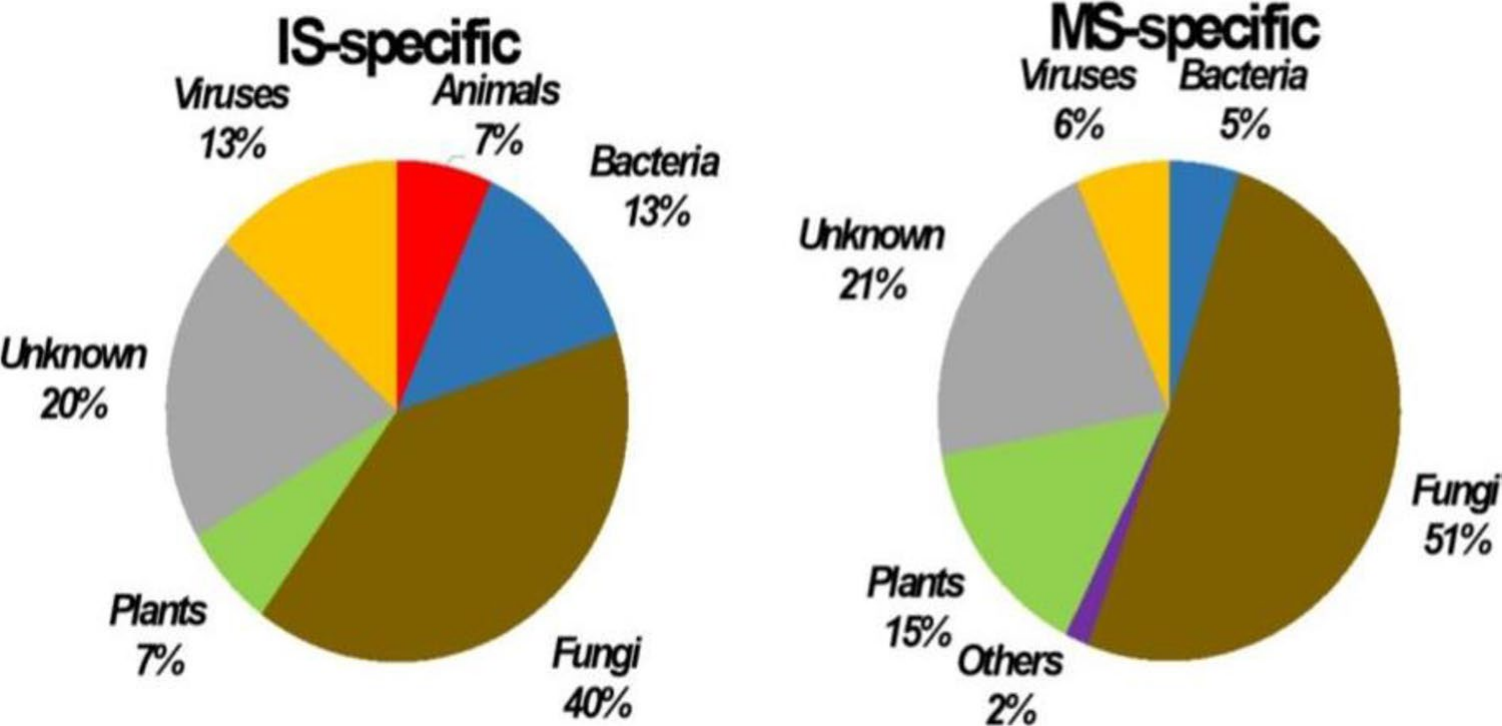
Taxonomic assignment of the most significant DEGs from Sonchus roots that were upregulated after intercropping with maize

Comparative analysis was performed through a two-step approach. First, we characterized genes that were present in only one group of libraries and that would be considered as specific for a given cultivation condition, prior to identification of differentially expressed genes (DEGs). This approach revealed a total of 168 unigenes that were further analyzed as 76 ‘specific’ genes and 92 DEGs (Supplementary Figure S4, Supplementary Table S6). Precisely, 15 unigenes were present in the IS libraries and were absent from the MS libraries (Table 1, Fig. 1). These unigenes were assigned to diverse organisms, including fungi (6), viruses (2), bacteria (2), nematodes (1), plant (1), and 3 unigenes were of unknown origin. The 6 fungal genes better matched sequences from Basidiomycetes and mainly encoded proteins involved in stress responses and defense, such as ROS-scavenging enzymes, heat shock proteins and stress responsive proteins (Table 1). The viral sequences showed high homology with the coat protein from lettuce big-vein virus. The plant unigene corresponded to a partial sequence of a Copia transposable element. No open reading frame (ORF) of significant length was deduced from the sequences of unknown origin.

**Table 1:** List of genes only expressed under intercropping or monoculture system. Descriptions were obtained through blastn and blastx searches. Descriptions followed by an asterisk (*) were obtained through searches in specific databases

Unigene	Closest organism	Best hit description	e value	Percent ident	Accession	KOG
Intercropping						
Unigene0002292	*Rhizoctonia solani*	CsbD domain-containing protein	2e − 57	89.00	ELU45956.1	V
Unigene0008170	*Artemisia annua*	LTR copia	5e − 53	58.62	PWA54489.1	AA
Unigene0018404	*Rhizoctonia solani*	Atg8 ubiquitin-like protein	9e − 79	99.15	EUC54649.1	R
Unigene0019995	*Rhizoctonia solani*	Heat shock protein	6e − 39	87.34	EUC58715.1	V
Unigene0030063	*Rhizoctonia solani*	Chaperone protein HSP31	3e − 123	78.54	CCO26766.1	V
Unigene0034376	*Rhizoctonia solani*	Glutathione peroxidase	1e − 95	86.54	CEL55554.1	V
Unigene0035424	*Cylicostephanus goldi*	Barrier to autointegration factor	5e − 42	73.03	VDN33281.1	V
Unigene0037935	*Lettuce big-vein virus*	Coat protein	0.0	89.69	MH356745.1	
Unigene0037937	*Lettuce big-vein virus*	Coat protein, complete cds	0.0	91.97	AB190527.1	
Unigene0053124		No hit				S
Unigene0055279		No hit				S
Unigene0055699		No hit				S
Unigene0058748	*Gemmatirosa kalamazoonesis*	23S ribosomal RNA	0.0	94.63	CP007128.1	
Unigene0058955	*Acinetobacter sp.*	Complete genome	0.0	99.75	CP051208.1	
Unigene0061510	*Ceratobasidium theobromae*	Lectin-like protein	2e − 71	76.55	KAB5590951.1	W
Monoculture						
Unigene0001104	*Lobosporangium transversale*	Acyl CoA binding protein	2e − 13	77.19	XM_022026596	I
Unigene0001107	*Rhizopus stolonife*	GPI-anchored membrane protein	1e − 16	52.38	RCI05082.1	M
Unigene0001231	*Endogone sp*	Heat shock protein Awh11/Hsp9	4e − 17	46.07	RUS21436.1	V
Unigene0001364	*Mortierella verticillata*	Secreted effector*	7e − 06	45.45	KFH71393.1	S
Unigene0001691	*Rhizophagus irregularis*	MD-2-related lipid recognition domain	0.0	99.42	XM_025310287.1	I
Unigene0002938		Secreted protein*				S
Unigene0003402	*Lactuca sativa*	Hydroxycinnamoyltransferase-like	6e − 87	79.03	XM_023883827.1	Q
Unigene0003926	*Choanephora cucurbitarum*	Peptidyl-prolyl cis–trans isomerase cyp5	1e − 76	71.86	OBZ86035.1	O
Unigene0004098	*Gigaspora rosea*	Plectin/S10 domain-containing protein	1e − 55	63.83	RIB07191.1	J
Unigene0005302	*Jimgerdemannia flammicorona*	Secreted protein*	6e − 44	52.63	RUP46267.1	S
Unigene0006605		No hit				S
Unigene0006820	*Absidia repens*	RNA-binding protein	4e − 17	64.64	XM_022022703.1	K
Unigene0007644		No hit				S
Unigene0009666		No hit				S
Unigene0012470	*Rhizophagus clarus*	Secreted effector*	7e − 16	40.38	GBB99714.1	S
Unigene0014068	*Lactuca sativa*	Basic blue protein-like	2e − 25	65.52	XP_023739420.1	C
Unigene0014088	*Helianthus annuus*	Copia	3e − 19	48.54	XP_022004759.1	AA
Unigene0014183	*Phycomyces blakesleeanus*	Translation elongation factor 1-alpha	0.0	85.35	AF157275.1	J
Unigene0015917	*Diversispora epigaea*	Secreted protein*	2e − 15	41.41	RHZ47330.1	S
Unigene0016248	*Jimgerdemannia flammicorona*	Secreted protein*	1e − 26	36.25	RUP46415.1	S
Unigene0016901	*Phycomyces blakesleeanus*	Biotin synthase	2e − 135	72.12	XM_018428819.1	H
Unigene0017952	*Jimgerdemannia flammicorona*	Universal stress protein UspA	5e − 47	66.67	RUP45634.1	V
Unigene0020339	*Phycomyces blakesleeanus*	Serine/threonine protein kinase	2e − 42	45.11	XP_018287438.1	T
Unigene0020914	*Jimgerdemannia flammicorona*	Hsp71-like protein	0.0	90.74	RUO96061.1	V
Unigene0021826	*Lactuca sativa*	Late embryogenesis abundant protein	1e − 64	70.79	XM_023905366.1	R
Unigene0022275	*Parasitella parasitica*	Reticulon-like protein	5e − 29	35.89	CEP15252.1	U
Unigene0022681		No hit				S
Unigene0025319	*Jimgerdemannia flammicorona*	Ctr copper transporter	9e − 27	62.16	RUS25062.1	P
Unigene0026357	*Rhizophagus irregularis*	Transmembrane protein	4e − 133	78.28	XM_025314841.1	S
Unigene0028252		No hit				S
Unigene0028515	*Chloroflexi bacterium*	Aminotransferase class V	7e − 52	53.07	NOZ73298.1	R
Unigene0028517	*Anaerolineae bacterium*	Aminotransferase class V	6e − 67	55.78	PWH12105.1	R
Unigene0028978	*Cynara cardunculus*	Cytochrome P450 81E8-like	2e − 157	83.30	XM_025116380.1	V
Unigene0030401	*Bifiguratus adelaidae*	Inorganic pyrophosphatase	2e − 140	77.17	OZJ06844.1	P
Unigene0033724	*Tanacetum cinerariifolium*	Copia	4e − 78	42.30		AA
Unigene0035150	*Potato virus T*	RNA-dependent RNA polymerase	1e − 93	51.80	BAM16482.1	
Unigene0036699	*Raspberry vein chlorosis virus*	Glycoprotein	2e − 22	64.67	MK257717.1	
Unigene0038532	*Grapevine leafroll-associated virus*	Gypsy	5e − 07	54.72	QGU17998.1	
Unigene0039942	*Lactuca sativa*	Subtilisin-like protease SBT1.7	1e − 69	77.32	XM_023891452.1	O
Unigene0040154	*Mortierella alpina*	Delta-9 fatty acid desaturase	4e − 165	72.39	AB015611.1	I
Unigene0040231	*Rhizophagus irregularis*	Secreted effector*	0.0	96.75	XM_025317364.1	S
Unigene0047718		No hit				S
Unigene0048596	*Helianthus annuus*	Ripening-related protein 1	4e − 138	86.19	XM_022147256.1	V
Unigene0050668	*Jimgerdemannia flammicorona*	BTG-domain-containing protein	3e − 49	51.04	RUP42730.1	D
Unigene0058766	*Lactuca sativa*	Uncharacterized protein	1e − 39	60.83	XP_023762598.1	S
Unigene0059083	*Potato virus T*	Replicase, movement protein	2e − 50	72.22	JQ394882.1	
Unigene0059194		No hit				S
Unigene0059513	*Rhizophagus irregularis*	Thioredoxin-like protein	4e − 92	75.40	XM_025311418.1	V
Unigene0059614	*Gemmatimonadetes bacterium*	3-ketoacyl-ACP reductase	6e − 40	43.06	PYP70173.1	I
Unigene0060200	*Gigaspora rosea*	Secreted effector*	1e − 20	41.54	RIB12644.1	S
Unigene0060271	*Lobosporangium transversale*	Ornithine aminotransferase	2e − 76	70.92	XM_022027200.1	E
Unigene0060285		No hit				S
Unigene0060739		Copia*				AA
Unigene0061463		Secreted protein*				S
Unigene0062269		No hit				S
Unigene0062564		Secreted protein*				S
Unigene0062959	*Phycomyces blakesleeanus*	TB2/DP1/HVA22-related protein	1e − 26	73.39	XM_018440061.1	V
Unigene0063164	*Rhizophagus irregularis*	Cytochrome b5	1e − 163	99.10	XM_025319531.1	C
Unigene0063327	*Phaeodactylum tricornutum*	Thioredoxin h	3e − 31	50.00	XP_002180660.1	V
Unigene0064008	*Diversispora epigaea*	Secreted effector*	6e − 20	45.54	RHZ53327.1	S
Unigene0064112	*Phycomyces blakesleeanus*	Phosphoadenosine reductase	2e − 52	68.39	XM_018429788.1	I

A total of 61 genes were present in MS libraries and absent from the IS libraries. These genes were mainly from fungi (31 sequences, 51%), whereas only 15% were from plants, while 21% could not be unambiguously assigned to a given organism (Table 1, Fig. 1). Fungal genes were all undoubtedly assigned to Glomeromycetes and corresponded to AMF inhabiting the Sonchus root system. Proteins were predicted from all fungal unigenes and were classified in various KOG categories, despite the moderate number of genes (Table 1, Fig. 1). The most represented categories were ‘trafficking, secretion and vesicular transport’ (8/31), followed by ‘stress response and defense’ (5/31) and lipid metabolism (4/31). This grouping was performed through manual annotation and identification of 8 putative secreted proteins, among which 5 were candidate effectors. In addition, annotation showed the presence of 3 putative secreted proteins and a Copia-like element among the open reading frames (ORFs) of unknown origin. Last, the eight sequences from plants were grouped to various KOG categories. Two genes encoded potential Copia-like elements. These findings indicate that intercropping induced an apparent transcriptional inactivation, if not extinction, of root-associated Glomeromycetes and promoted development of Basidiomycetes activating responses to stress.

**Fig. 2:**
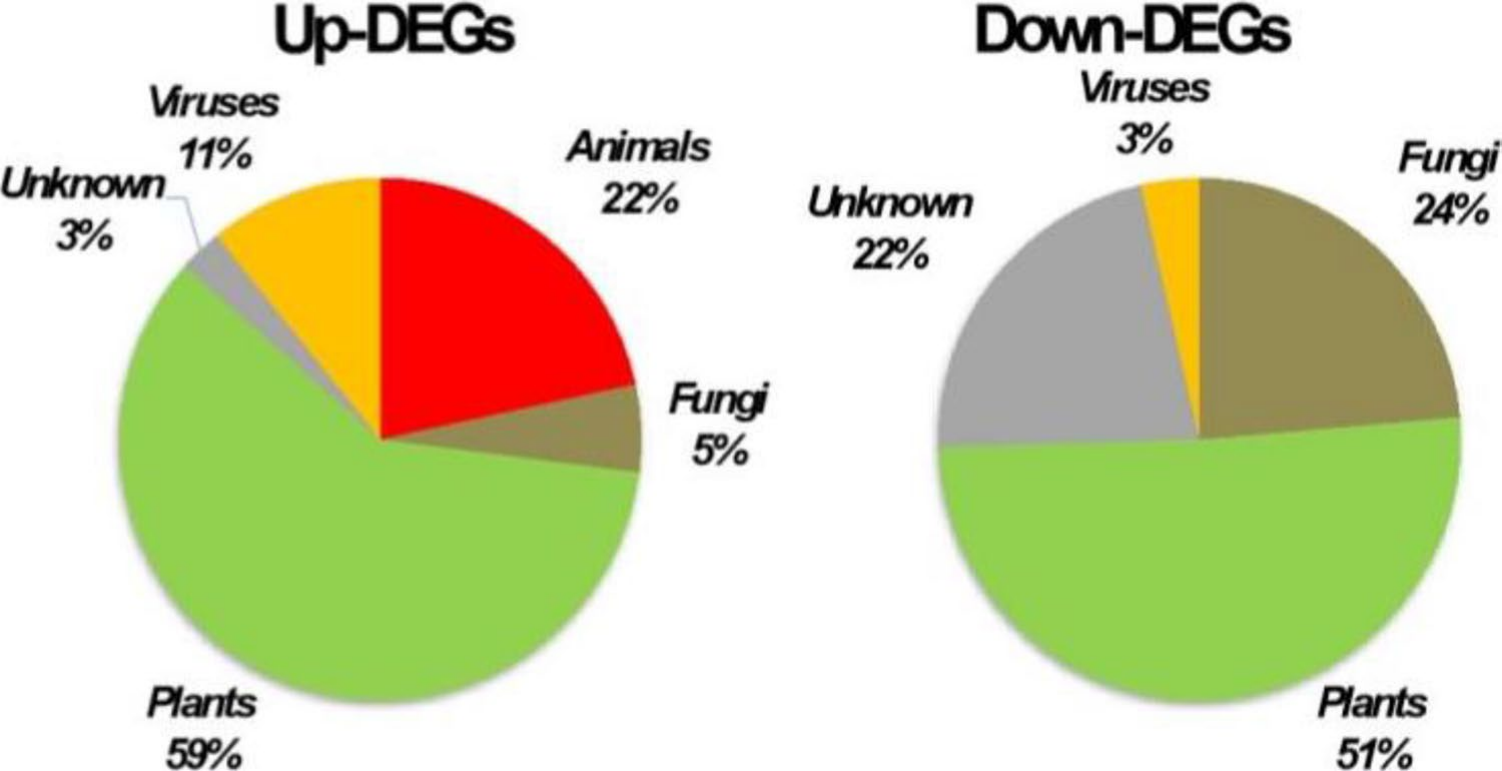
Analysis of Sonchus root transcripts that are downregulated upon intercropping with maize. **A**: Taxonomic assignment of DEGs that were present in MS libraries and absent from IS libraries. **B**: Functional annotation of genes of potential fungal origin based on the Eukaryotic orthologous group (KOG) classification. Transposable elements constitute an additional category (AA). **C**: Taxonomic assignment of DEGs that were downregulated upon intercropping. **D**: KOG classification of the unigenes from plants

Analysis showed that 37 DEGs were upregulated upon intercropping and 55 DEGs were downregulated (Table 2, Fig. 2). DEGs were mainly of plant (59%) and animal (22%) origin, whereas viruses and fungi were less represented (Fig. 2). Plant sequences corresponded to fragments of ribosomal DNA (rDNA) or mitochondrial DNA (mtDNA), despite carrying out a purification step of mRNA during generation of libraries. Sequences from mtDNA mainly encoded various subunits of the NADH dehydrogenase complex, suggesting an enhanced aerobic activity (Table 2). All unigenes from possible animal origin matched nematode sequences and were derived from ribosomal RNA, with exception of an unigene encoding a putative secreted effector of *M. incognita* which is over-expressed in parasitic stages of the nematode (Nguyen et al., 2018). Viral sequences mainly encoded RNA polymerase. The two fungal candidates were assigned to basidiomycetes. One of the fungal sequences encoded a potential membrane permease, whereas the other was derived from ribosomal DNA. So, most upregulated unigenes upon intercropping might constitute ribosomal and mitochondrial contaminations of the mRNA preparations used to generate RNA-Seq libraries.

**Table 2:** List of differentially expressed genes (DEGs) identified after Maize-Sonchus intercropping. Descriptions were obtained based on blastn and blastx searches. Descriptions followed by an asterisk (*) were based on additional searches in specific databases

Unigene	log2 ratio (ISR/MSR)	Closest organism	Description	e value	Percent ident	Accession	KOG
Unigene0038517	11.39	*Lettuce big-vein virus*	Coat protein	0.0	88.96	AB114138.1	
Unigene0055277	11.39	*Beihai picorna-like virus 80*	RNA-dependent RNA polymerase	1e − 99	34.52	APG76683.1	
Unigene0055278	9.98	*Beihai picorna-like virus 80*	RNA-dependent RNA polymerase	1e − 101	34.71	APG76683.1	
Unigene0058585	8.37	*Meloidogyne incognita*	Small subunit ribosomal RNA gene	0.0	100.00	MK292132.1	
Unigene0056709	8.22	*Meloidogyne hapla*	28S ribosomal RNA gene	0.0	97.56	KU180679.1	
Unigene0058589	7.90	*Meloidogyne incognita*	Large subunit ribosomal RNA gene	0.0	98.33	MT406772.1	
Unigene0045083	7.87	*Meloidogyne arenaria*	28S ribosomal RNA gene,	1e − 160	98.48	AF023854.1	
Unigene0054767	6.88	*Lactuca sativa*	Mitochondrion, complete genome	0.0	98.86	NC_042756.1	
Unigene0026950	6.82	*Rhizoctonia solani*	Membrane permease	3e − 139	87.44	KDN38622.1	P
Unigene0056235	5.42	*Meloidogyne arenaria*	Small subunit ribosomal RNA gene	0.0	100.00	MT329687.1	
Unigene0056955	5.37	*Meloidogyne arenaria*	5S, 18S, 5.8S and 28S ribosomal RNA	0.0	98.84	U42342.1	
Unigene0058967	5.31		No hit				
Unigene0058879	4.74	*Meloidogyne oryzae*	Complete ribosomal DNA cluster	0.0	92.97	LS974441.1	
Unigene0058968	4.73	*Nematode virus*	RNA-dependent RNA polymerase	2e − 120	27.04	YP_009342285.1	
Unigene0049525	4.69	*Paraprenanthes diversifolia*	NADH dehydrogenase subunit 1	0.0	97.41	MN661146.1	C
Unigene0058885	4.45	*Lactuca sativa*	26S ribosomal RNA gene	0.0	96.71	KT179738.1	
Unigene0058883	4.44	*Taraxacum officinale*	Small subunit ribosomal RNA gene	0.0	98.54	KY860926.1	
Unigene0058874	4.43	*Lygodesmia juncea*	26S ribosomal RNA gene	0.0	99.76	KT179737.1	
Unigene0058880	4.40	*Tecomaria capensis*	External transcribed spacer	9e − 159	100.00	MK678758.1	
Unigene0058877	4.37	*Diplostephium foliosissimum*	External transcribed spacer	0.0	99.32	KX064035.1	
Unigene0058869	4.35	*Solanum pinnatisectum*	28S ribosomal RNA gene	0.0	98.23	CP047566.1	
Unigene0057605	4.31	*Paraprenanthes diversifolia*	NADH dehydrogenase subunit 1	0.0	98.84	MN661146.1	C
Unigene0058875	4.27	*Adenocalymma ubatubense*	28S ribosomal RNA	0.0	97.59	MK678746.1	
Unigene0058871	4.25	*Lactuca ludoviciana*	26S ribosomal RNA gene	0.0	99.76	KT179738.1	
Unigene0058884	4.25	*Thanatephorus cucumeris*	25S large subunit ribosomal RNA gene	4e − 165	92.20	DQ917658	
Unigene0058872	4.22	*Taraxacum officinale*	Small subunit ribosomal RNA gene	1e − 151	98.11	KY860926.1	
Unigene0058878	4.20	*Malus baccata*	External transcribed spacer	0.0	98.11	MN215980.1	
Unigene0056705	4.16	*Lactuca sativa*	NADH dehydrogenase subunit 5	0.0	97.41	NC_042756.1	C
Unigene0058873	4.15	*Bidens hillebrandiana*	Small subunit ribosomal RNA gene	0.0	99.80	KY860926.1	
Unigene0058636	3.89	*Paraprenanthes diversifolia*	NADH dehydrogenase subunit 1	0.0	98.56	MN661146.1	C
Unigene0049522	3.86	*Lactuca serriola*	Mitochondrial large subunit ribosomal RNA	0.0	99.82	NC_042378.1	
Unigene0054786	3.70	*Paraprenanthes diversifolia*	ATPase subunit 4	0.0	97.97	MN661146.1	C
Unigene0055915	3.66	*Lactuca sativa*	ATPase subunit 8 + Cox3	0.0	98.76	NC_042756.1	C
Unigene0049553	3.30	*Meloidogyne incognita*	Candidate secreted effector Minc04729 mRNA	5e − 17	92.75	KX907752.1	V
Unigene0055355	3.03	*Paraprenanthes diversifolia*	NADH dehydrogenase subunit 4	0.0	99.05	MN661146.1	C
Unigene0056410	2.93	*Cynara cardunculus*	LTR copia	0.0	83.83	XM_025109257.1	AA
Unigene0049906	2.70	*Paraprenanthes diversifolia*	Small subunit ribosomal RNA	0.0	99.37	MN661146.1	
Unigene0040688	-2.49	*Lactuca sativa*	UDP-glucose 4-epimerase	0.0	83.40	XM_023890365.1	G
Unigene0061632	-2.76	*Lactuca sativa*	EG45-like domain containing protein	4e − 123	86.05	XM_023875967.1	V
Unigene0054384	-3.08	*Lactuca sativa*	Senescence-specific cysteine protease	0.0	90.43	XM_023886231.1	V
Unigene0060210	-3.09	*Lactuca sativa*	Blue copper protein-like	0.0	89.71	XM_023908807.1	C
Unigene0059337	-3.25		No hit				S
Unigene0032094	-3.27	*Lactuca sativa*	Uncharacterized LOC111879552	0.0	83.75	XM_023876017.1	S
Unigene0006984	-3.32	*Lactuca sativa*	Secreted protein	0.0	88.95	XM_023903228.1	S
Unigene0019785	-3.50	*Lactuca sativa*	Amino acid permease 4-like	0.0	92.45	XM_023915076.1	E
Unigene0009094	-3.68	*Lactuca sativa*	NRT1/ PTR FAMILY 5.2-like	0.0	89.58	XM_023891618.1	P
Unigene0006870	-3.89	*Lactuca sativa*	CASP-like protein 1E2	0.0	87.14	XM_023912887.1	M
Unigene0014390	-4.13	*Cynara cardunculus*	GDSL esterase/lipase LTL1-like	0.0	80.60	XM_025122686.1	M
Unigene0009135	-4.14	*Lactuca sativa*	Expansin-A15-like	0.0	85.98	XM_023874274.1	W
Unigene0054293	-4.15	*Lactuca sativa*	Subtilisin-like protease	0.0	86.62	XM_023872856.1	O
Unigene0062310	-4.36	*Lactuca sativa*	Oleosin-like	2e − 158	79.91	XM_023879394.1	I
Unigene0019281	-4.60	*Lactuca sativa*	Late embryogenesis abundant protein D-34-like	0.0	84.53	XM_023888992.1	V
Unigene0056198	-4.65	*Lactuca sativa*	Transposase	2e − 51	34.88	XP_023771188.1	AA
Unigene0010666	-4.77	*Cynara cardunculus*	Cell number regulator 10-like	2e − 79	73.66	XM_025136183.1	V
Unigene0043499	-4.78	*Rhizophagus irregularis*	Aquaporin 3	8e − 78		XM_025310287.1	P
Unigene0058329	-4.88		Harbinger*				AA
Unigene0045534	-4.98		SEC7 family protein*				S
Unigene0061629	-5.24		Secreted protein*				S
Unigene0050192	-5.33		No hit				S
Unigene0026816	-5.34	*Lactuca sativa*	Nucleoredoxin 2	0.0	87.45	XM_023910071.1	R
Unigene0032769	-5.43	*Drechmeria coniospora*	MD-2-related lipid-recognition domain protein	7e − 13	35.51	KYK59549.1	I
Unigene0055249	-5.47	*Lactuca sativa*	Gypsy	4e − 119	54.72	CAB4088913.1	AA
Unigene0040379	-5.57	*Lactuca sativa*	NRT1/ PTR FAMILY 6.2-like	0.0	89.03	XM_023889421.1	P
Unigene0030689	-5.71	*Colletotrichum higginsianum*	Phosphate:H + symporter	5e − 58	71.12	XM_018296238.1	P
Unigene0020641	-5.75		Nuclear export signal containing-protein*				S
Unigene0036142	-5.79	*Sodiomyces alkalinus*	60S Acidic ribosomal protein	2e − 40	75.35	XM_028607766.1	J
Unigene0058581	-5.85		No hit				S
Unigene0063074	-5.90	*Zea mays*	Calmodulin	7e − 20	75.29	EU958417.1	T
Unigene0040146	-5.92	*Rasamsonia emersonii*	Autophagy-related protein 8 precursor	1e − 46	73.75	XM_013467937.1	R
Unigene0062868	-6.15	*Calocera cornea*	Heavy metal transport/detoxification protein	8e − 22	72.31	KZT58956.1	V
Unigene0058582	-6.24		hAT*				AA
Unigene0013756	-6.35		Secreted protein*				S
Unigene0035420	-6.38		No hit				S
Unigene0035325	-6.44	*Tanacetum cinerariifolium*	Peptidyl-prolyl cis–trans isomerase CYP21-4	6e − 13	60.29	GEU91633.1	O
Unigene0013755	-6.48	*Trematosphaeria pertusa*	Actin cytoskeleton protein-like protein	1e − 156	78.88	XM_033822720.1	Z
Unigene0014673	-6.52	*Phycomyces blakesleeanus*	Actin-binding protein	4e − 38	70.13	XM_018429765.1	Z
Unigene0000471	-6.67	*Gloeophyllum trabeum*	Cytochrome b5	2e − 30	73.94	XM_007867963.1	C
Unigene0035926	-6.77		No hit				S
Unigene0046621	-6.77	*Botrytis virus F*	Replicase	5e − 77	37.60	NP_068549.1	
Unigene0010451	-6.82	*Tilletiopsis washingtonensis*	GTP-binding protein SAR1	7e − 83	72.87	XM_025743395.1	T
Unigene0019201	-6.84	*Lactuca sativa*	Reticuline oxidase-like	3e − 134	86.55	XM_023909137.1	Q
Unigene0044498	-6.87	*Lactuca sativa*	Chaperone for superoxide dismutase	3e − 93	83.04	XM_023894804.1	V
Unigene0021697	-6.89	*Lactuca sativa*	Aquaporin NIP2-1	0.0	86.52	XM_023913024.1	P
Unigene0058584	-7.03	*Botrytis virus F*	Replicase	1e − 31	36.17	NP_068549.1	
Unigene0028090	-7.04	*Lactuca sativa*	Subtilisin-like protease	0.0	91.42	XM_023891500.1	O
Unigene0010575	-7.05	*Lactuca sativa*	2-oxoglutarate-dependent dioxygenase	0.0	88.75	XM_023892765.1	E
Unigene0017475	-7.19		No hit				S
Unigene0022011	-7.27	*Trametes versicolor*	ADP-ribosylation factor GTPase	8e − 120	83.78	XM_008037236.1	T
Unigene0061600	-7.40	*Schizophyllum commune*	Glycoside hydrolase family 18 protein	6e − 17	67.63	XM_003031731.1	M
Unigene0045061	-7.85	*Lactuca sativa*	Transmembrane protein	0.0	83.87	XM_023876019.1	S
Unigene0024154	-8.44	*Wallemia ichthyophaga*	CsbD domain-containing protein	2e − 11	45.16	TIB42155.1	V
Unigene0052035	-9.10	*Artemisia annua*	Copia	0.0	52.79	PWA62550.1	AA

Analysis of the DEGs that were downregulated upon intercropping showed that most were plant genes (28/55, 51%), whereas fungal candidates and unassigned sequences contributed to 24% and 22% of the sample, respectively (Fig. 2). DEGs of possible plant origin were classified into diverse KOG categories, and the findings showed that stress response and defense, protein turnover, transport, signal transduction and transposable elements constituted were the most represented categories (Table 2). Analysis of the 13 genes of likely fungal origin showed that 4 unambiguously originated from ascomycete and mucoromycete donors; however, the other genes did not display signatures of a particular fungal lineage. Fungal candidate genes encoded proteins which were classified into various categories, including stress response, transport, cytoskeleton and signal transduction. Notably, only a limited number of genes were identified, so that no clear functional trend was thus deduced from this annotation. Six out of the 12 DEGs of unknown origin comprised ORFs that encoded putative secreted proteins including a protein containing a nuclear export signal, a Sec7 family protein, as well as 3 potential transposable elements. Therefore, 6 DEGs that remained had no taxonomic assignment and functional annotation (Table 2).

To validate the findings obtained from RNA-Seq data, 14 genes were randomly selected to confirm the observed effect of intercropping by quantitative real time (qRT)-PCR. Genes that were upregulated were not selected because they were mainly derived from ribosomal and mitochondrial RNA, and were mainly contaminants from mRNA preparations. The selected genes encoded various enzymes whose expression was downregulated as shown in RNA-Seq experiments. A significant positive correlation between RNA-Seq and qRT-PCR data was observed for 11/14 genes (*p* value < 0.05) (Supplementary Figure S5). Notably, the samples for qRT-PCR experiments were the same as those used for RNA-Seq analysis.

## Discussion

We show in the present study that the excluder-to-hyperaccumulator switch and improved remediation properties of *S. asper* resulting from intercropping do not rely on significant modifications of the plant transcriptome, but rather on changes in composition of root-associated microbiome.

Analysis of RNA-Seq data and comparison of RNA libraries from Sonchus roots grown in monoculture with those grown under intercropping showed significant changes in only 1% (168/16878) of the initial unigene dataset. We noted that the relative abundance of the major taxonomic categories was significantly different across the total dataset and the set of genes submitted to transcriptional modifications. Notably, genes of plant origin, which comprised > 98% of the total unigene assembly, were under-represented in the differential set, indicating that intercropping does not induce changes in the plant transcriptome. However, intercropping maize significantly affects the transcriptome of associated fungi and viruses.

Upregulated DEGs after intercropping mainly originated from ribosomal and mitochondrial RNA, with different organisms of origin. These sequences were considered as contamination of the initial mRNA preparations and thus should have been discarded from further analyses. Hence, it was not possible to validate their increased abundance upon intercropping by qRT-PCR. Yet, their over-representation reflects an increased biomass of the organisms from which they originate. This is consistent with previous findings, and the confirmation provided by the current study that intercropping stimulates Sonchus development (Pu et al., 2018). In addition, increased development of the root system likely provides a favorable niche to various components of associated rhizosphere. Notably, de novo assembly of RNA-Seq reads and further taxonomic assignments revealed unigenes that displayed a best hit against Genbank entries from animals, including chordates and arthropods. This finding can be attributed to presence of traces of insects, rodents and other small animals at the vicinity of Sonchus roots grown in farmland soils. In addition, we can deduce the presence of nematodes, which are major plant pathogens that attack a myriad of plant families, including Sonchus in farmland soils (Jones et al. 2013). The presence of nematode-derived sequences among upregulated DEGs may also indicate an increased plant susceptibility to root pathogens, which may be caused by the observed decreased responses of the root tissues to stress.

Over-representation of fungal genes was further analyzed because different phyla occur in different situations. Basidiomycetes were identified among sequences derived from the intercropping system, whereas Ascomycetes and Glomeromycetes which showed significant abundance in monoculture were absent in IS libraries. Further studies should explore the root system of Sonchus grown under monoculture and under intercropping to verify whether intercropping Sonchus and maize leads to a transcriptional inactivation of these fungi or causes their extinction. Sonchus roots are inhabited by diverse fungal communities comprising both dark septate endophytes (DSE) and arbuscular mycorrhizal fungi (AMFs), which belong to Ascomycetes and Glomeromycetes, respectively (Pendleton and Smith 1983; Knapp et al. 2012). These phyla were identified from the taxonomic assignment of unigenes present in monoculture and were not present in the intercropping libraries. So, their possible extinction, associated to the increase in abundance of basidiomycetes akin to the plant pathogen *Rhizoctonia solani*, as well as pathogenic nematodes, which may have increased owing to the diminished expression of plant responses to stress, suggests that intercropping indirectly leads to increased plant susceptibility to pests. Moreover, the increased phytoextraction efficiency that we observed, resulting from intercropping, may cause a reduced toxicity of the soil, which offers more favorable conditions for re-shuffling of biotic interactions and a new composition of the root-associated microbiome.

The excluder-to-hyperaccumulator modified characteristics of Sonchus under intercropping system were mainly attributed to increased Pb translocation factor that resulted from the concomitant increase in Pb concentration in shoots and a decrease in roots. These findings and the observed significant decrease of effective Pb in the rhizosphere soil (Pu et al., 2018) may suggest that the excluder characteristics of Sonchus towards lead rely on its endophytic fungal community. Consequently, intercropping possibly dissociates these biotic interactions, thus restoring a basic ‘accumulator phenotype’ previously reported for other heavy metals such as cadmium (Pu et al., 2018). This hypothesis is based on several observations. First, the structures formed by DSEs and AMFs may constitute a physical barrier that absorbs and retains heavy metals and consequently leads to reduced root-to-shoot metal translocation, as a potential protection strategy of shoot tissues against metal damage (Zhan et al. 2019). This is suggested by the relatively high abundance of Glomeromycete genes in MS libraries. Second, a significant proportion of these genes encode secreted proteins including potential effectors, which are secreted by pathogenic microorganisms to defeat plant defense mechanisms and manipulate cellular functions of their hosts to achieve infection (Khan et al., 2018). Moreover, these proteins are used by symbiotic fungi for successful plant colonization (Kamel et al., 2017). Absence of these genes in the IS libraries or among the upregulated DEGs indicates symbiosis disruption and consequent activation (or suppression of the inhibition) of root-to-shoot translocation of Pb in a transcription-independent manner. Suppression of endophytic fungi may also explain the global decrease in lead concentration in Sonchus roots under intercropping system.

If hyperaccumulator characteristics of Sonchus are actually caused by suppression of endophytic fungi after intercropping, then maize may be responsible in several ways. Hence, intercropping may induce attraction and subsequent migration of the fungal community from Sonchus towards maize roots. In addition, endophytes may be eliminated by Sonchus through a decreased tolerance of the plant to its associated microbiota. We can also suppose interactions between the Sonchus-associated microbiome and the biotic community associated with maize roots and establishment of antagonistic interactions among these different microorganisms which may have caused a decrease or suppression of Sonchus endophytes. Last, maize root exudates contain molecules that may be toxic to Sonchus-associated fungi or can destabilize the symbiotic associations. Several studies explored composition of maize root exudates and reported that benzothiazole (BZO), an aromatic heterocyclic compound, and benzoxazinoids, such as 2,4-dihydroxy-7-methoxy-1,4-benzoxazin-3-one (DIMBOA) and its degradation product 6-methoxy-benzoxazolin-2(3H)-one (MBOA), have antimicrobial activity against plant pathogenic fungi and oomycetes (Yang et al. 2014; Mei et al. 2019). Other molecules such as phenolic acids, present in root exudates, have a strong antimicrobial activity against these microbial pathogens (Zhu and Morel 2019; Zhang et al. 2020). Therefore, maize can be used as a good helper to improve remediation abilities of hyperaccumulator *S. asper* through intercropping. In addition, identifying the components of maize root exudates and assessing their potential antifungal activity against endophytes and AMFs of Sonchus can provide a basis on exploring the mechanisms described in the present study.

A risk is that the use of edible plants for phytoremediation purposes can increase the possibilities of biomagnification of heavy metals into food chain. However, maize tolerance to metals significantly varies from one variety to another. In the present study, a variety that accumulates metals at a low grade was selected, and intercropping resulted in a reduced uptake of lead in both aerial and above-ground tissues. This finding is consistent with a previous study (Ji et al., 2017). In addition, intercropping of hyperaccumulators with edible plants is a widely developed strategy that enhances phytoremediation without affecting crop production or quality (Deng et al. 2016; Yang et al. 2021; Zou et al. 2021). Therefore, intercropping *Sedum plumbizincicola* in wheat growth season under wheat-rice rotation can improve phytoremediation of heavy metal-contaminated soil and decrease the food chain risk of rotated rice (Zhao et al. 2011). Moreover, *S. plumbizincicola* intercropped with maize at an appropriate planting density can achieve high remediation efficiency of contaminated soil without affecting productivity of maize crop (Deng et al. 2016). These findings indicate that intercropping with edible plants can be used in agriculture and management of environment for phytoremediation purposes.

## Conclusion

We report an ‘excluder-to-hyperaccumulator’ mode transition of *Sonchus asper* towards lead when intercropped with maize. However, the fundamental mechanisms underlying the improved phytoremediation efficiency of Sonchus have not been fully elucidated. We show here that intercropping-induced shifts among plant-associated microbiota communities result in a ‘root-to-shoot’ transition of lead transportation and sequestration. Moreover, maize root-derived exudates may play an important (positive and negative) role in reshaping this microbial community. Our study provides a new conceptual framework for further studies of plants and biotic relationships to elucidate mechanisms underlying heavy metal tolerance and remediation of polluted soils. The findings also confirm that combined microbiome-assisted bioremediation with intercropping is a valuable method for remediation of contaminated soils.

## Supplementary Information

Below is the link to the electronic supplementary material.

Supplementary file1 (PPTX 1735 KB)

Supplementary file2 (DOCX 22 KB)

## Data Availability

Not applicable.
