# Supplementary material for: Root-associated microbiota drive phytoremediation strategies to lead of Sonchus Asper (L.) Hill as revealed by intercropping-induced modifications of the rhizosphere microbiome
Source: Environ Sci Pollut Res Int. 2021 Nov 19;29(16):23026–40. doi: 10.1007/s11356-021-17353-1 (PMC8979924; doi:10.1007/s11356-021-17353-1)
Supplement: Supplementary file 1 — Supplementary file1 (PPTX 1735 KB) [file 11356_2021_17353_MOESM1_ESM.pptx]

## Slide 1
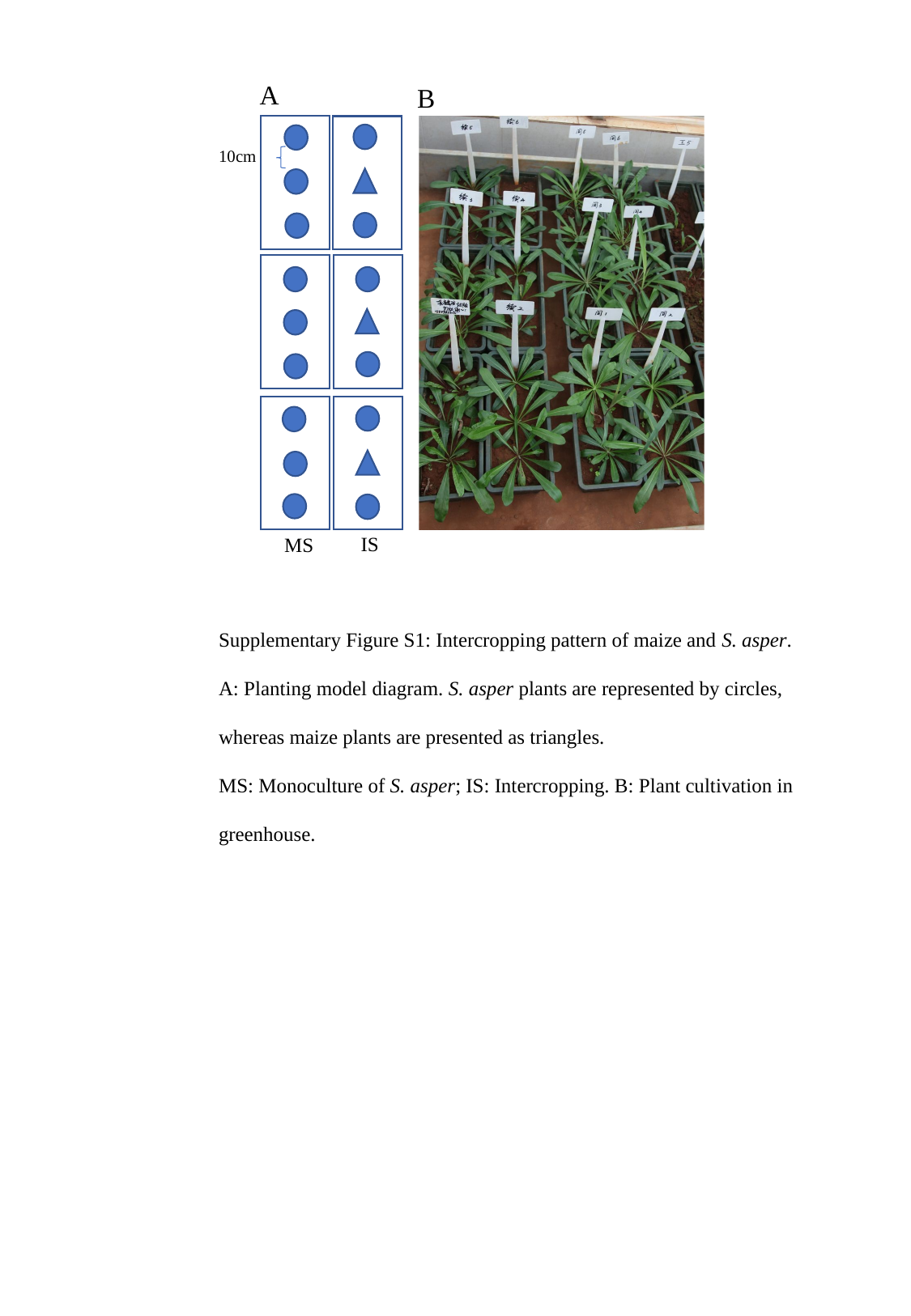

A
B
10cm
IS
MS
Supplementary Figure S1: Intercropping pattern of maize and S. asper.
A: Planting model diagram. S. asper plants are represented by circles, whereas maize plants are presented as triangles.
MS: Monoculture of S. asper; IS: Intercropping. B: Plant cultivation in greenhouse.

## Slide 2
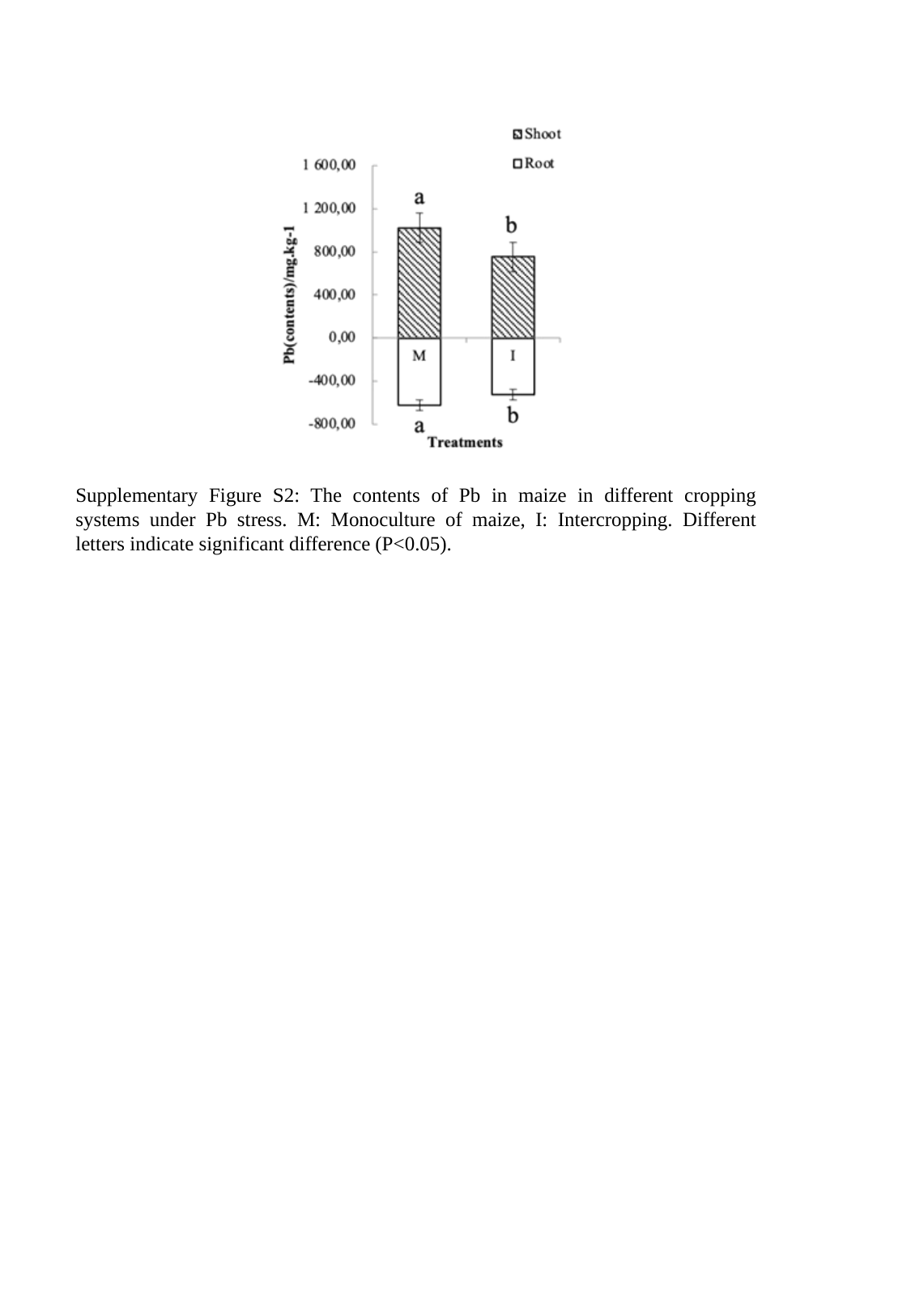

Supplementary Figure S2: The contents of Pb in maize in different cropping systems under Pb stress. M: Monoculture of maize, I: Intercropping. Different letters indicate significant difference (P<0.05).

## Slide 3
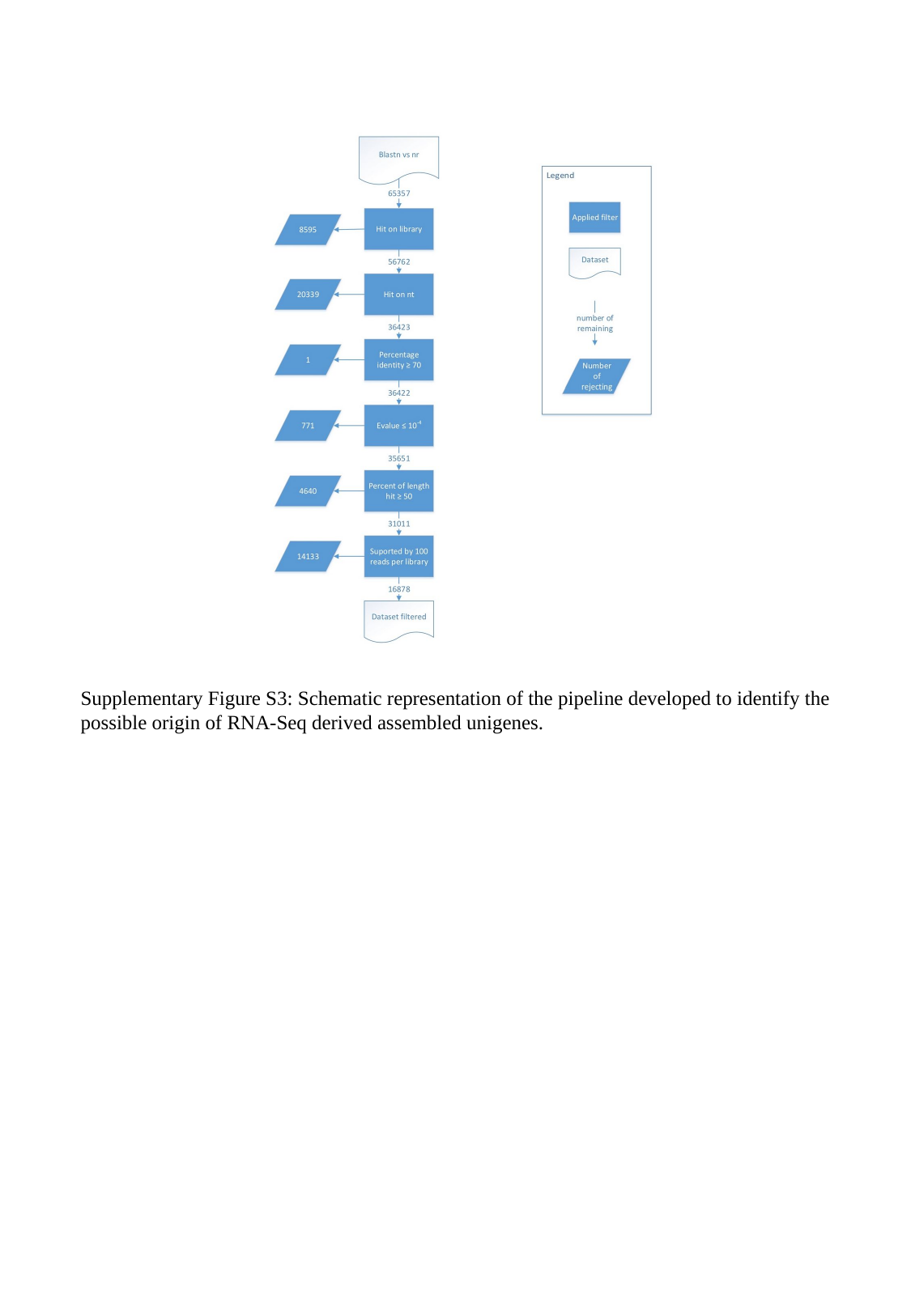

Supplementary Figure S3: Schematic representation of the pipeline developed to identify the possible origin of RNA-Seq derived assembled unigenes.

## Slide 4
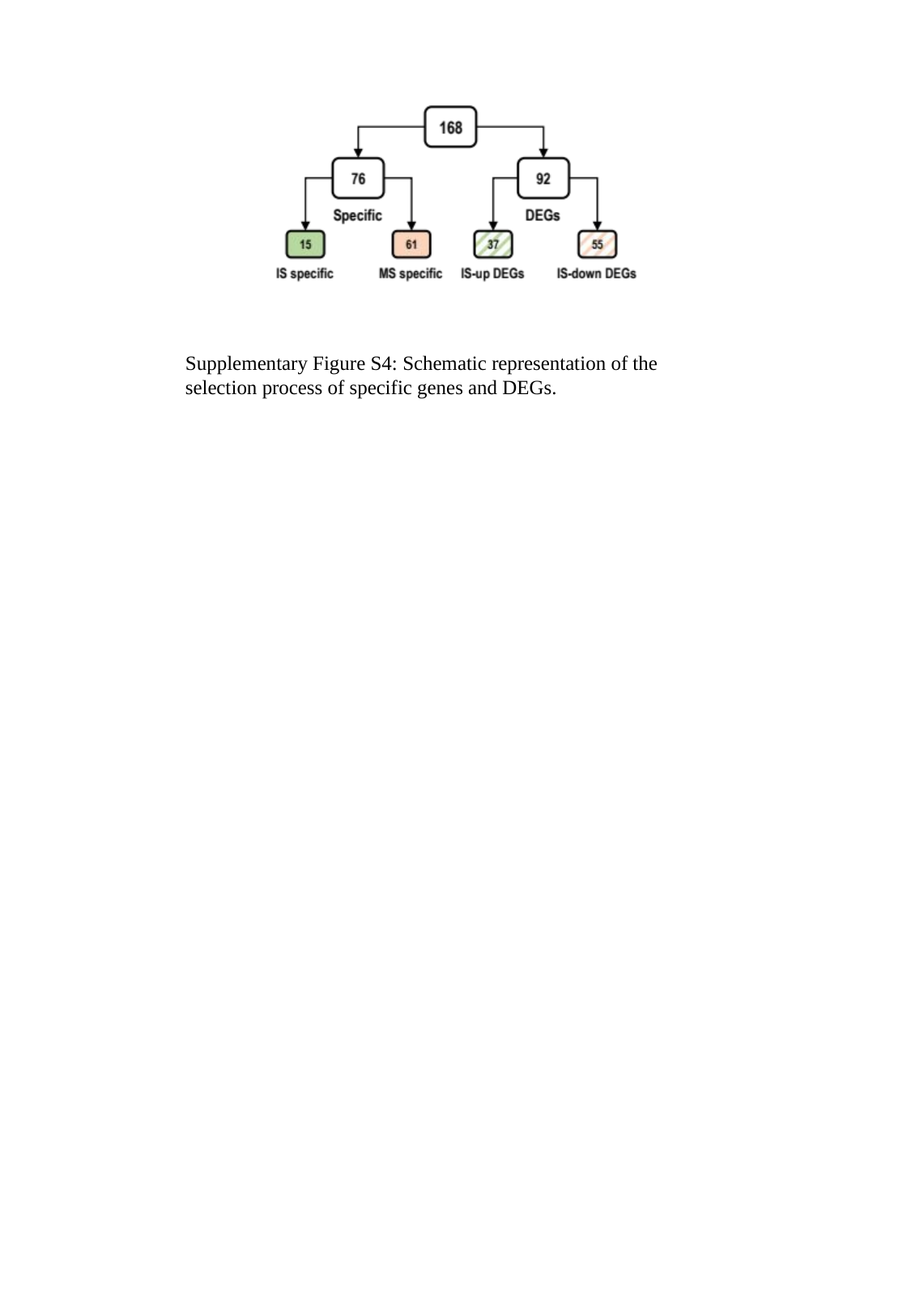

Supplementary Figure S4: Schematic representation of the selection process of specific genes and DEGs.

## Slide 5
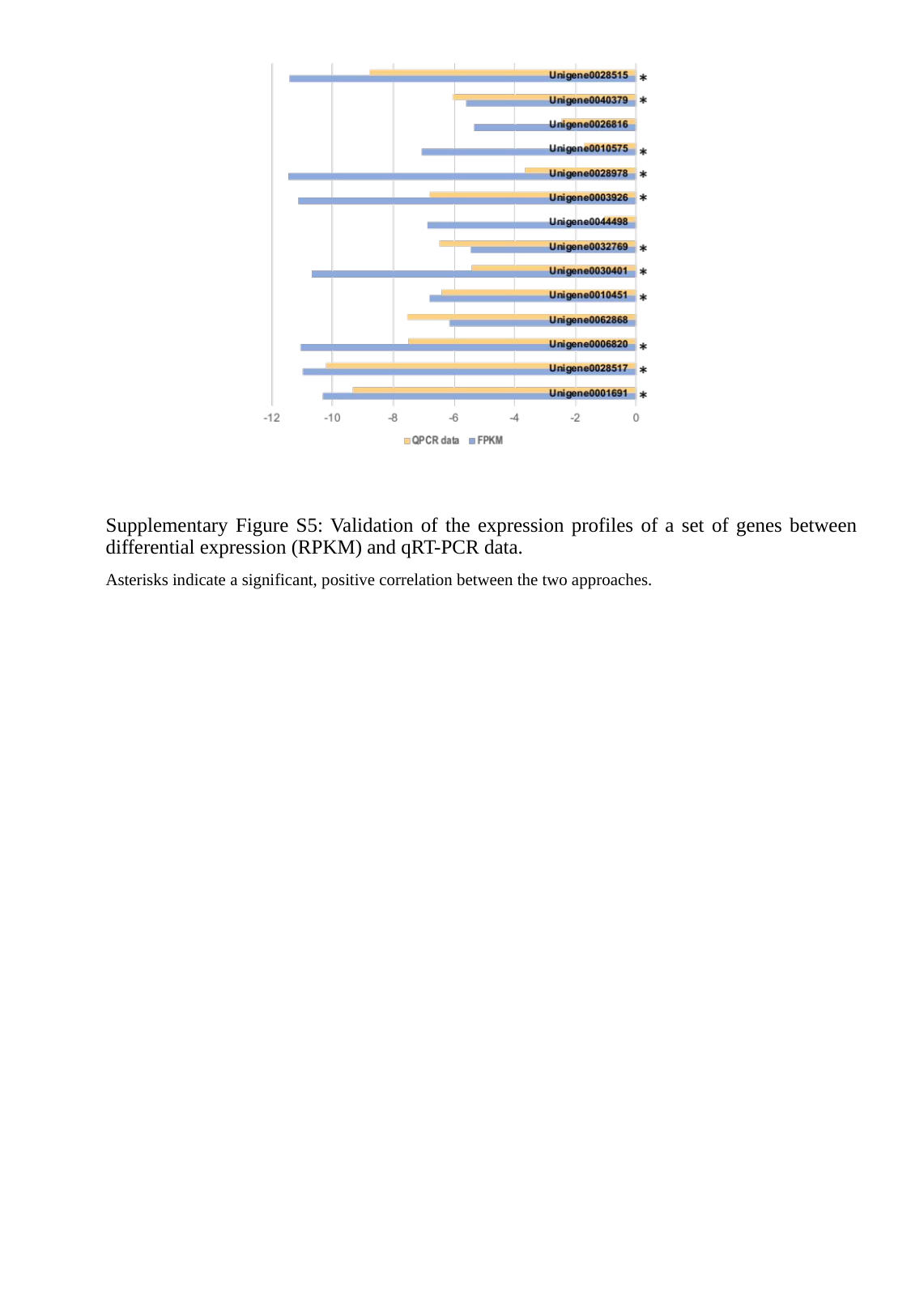

Supplementary Figure S5: Validation of the expression profiles of a set of genes between differential expression (RPKM) and qRT-PCR data.
Asterisks indicate a significant, positive correlation between the two approaches.
