# Supplementary material for: Root-associated microbiota drive phytoremediation strategies to lead of Sonchus Asper (L.) Hill as revealed by intercropping-induced modifications of the rhizosphere microbiome
Source: Environ Sci Pollut Res Int. 2021 Nov 19;29(16):23026–40. doi: 10.1007/s11356-021-17353-1 (PMC8979924; doi:10.1007/s11356-021-17353-1)
Supplement: Supplementary file 2 — Supplementary file2 (DOCX 22 KB) [file 11356_2021_17353_MOESM2_ESM.docx]

Supplementary Table S1: Biomass (g plant^-1^) of *S. asper* grown in monoculture and through intercropping with maize.

| **Cropping system** | **Shoots** | **Roots** |
| --- | --- | --- |
| Monoculture | 1.40±0.11b | 0.43±0.03b |
| Intercropping | 2.0±0.15a | 0.53±0.05a |

Data are means ± SD (n=3). Values with different letters are significantly different（P<0.05).

Supplementary Table S2: Pb content and translocation factor of S. asper grown in monoculture and through intercropping with maize

| **Cropping system** | **Pb content in shoot (mg kg-1)** | **Pb content in root (mg kg-1)** | **Translocation factor** |  |
| --- | --- | --- | --- | --- |
|  |  |  |  |  |
| Intercropping | 1482.42 ± 249.48a | 825.62 ± 78.97b | 1.795 |  |
| Monoculture | 997.92 ± 44.44b | 1195.01 ± 165.19a | 0.835 |  |

Data are means ± SD (n=3). Values with different letters are significantly different（P<0.05).

Supplementary Table S3: Primers used in the study.

| Primer | Sequence 5' > 3' |
| --- | --- |
| actin F | ACCATCTCCAGAATCCAGCAC |
| actin R | AGCACCTCTAAATCCCAAAGC |
| 10451 F | CCAAACAACGGGAAAGGG |
| 10451 R | CGACGCCACAGAGCACATA |
| 10575 F | GGTTGATGAAAAAACTCCCTCC |
| 10575 R | CTAAGGCGTGTCGTGAATGG |
| 1691 F | TGCGAACCATTTCCCAGAT |
| 1691 R | ATGCTCCATACCCTTTCCACTA |
| 26816 F | CATGCTTCTCCTTTTCTTGCC |
| 26816 R | TTGCGATTCCGTATGCTGAT |
| 28515 F | AGAATGAATGAAATCGTGCCTC |
| 28515 R | CGTCGGCTACCTCCTCAAA |
| 28517 F | GGATGCGTTACTGGTGAGGAC |
| 28517 R | CCTTATCCATCGCCACACG |
| 28978 F | TGGGCTTTGTCACTTCTGCTA |
| 28978 R | TCCGACTCATCCACAATACGA |
| 30401 F | CACCAAGGTCGAAAATCAAAAC |
| 30401 R | GCATCATCAACGAGCCCAC |
| 32769 F | GACGGTCGTGTTGGAAAGC |
| 32769 R | AAACGAGTGGTTCCGCATCT |
| 3926 F | TCAGGGAGTGAAGACCGAGC |
| 3926 R | GGGCATCATCTTCGTCGTC |
| 40379 F | GCAAGTAAGCAGTATGGCAAG |
| 40379 R | GTTTTGGACCGCTTATGCT |
| 44498 F | GCTCCGTTGTTCCAATCCA |
| 44498 R | GCTGGAGAAACCACCAAACAC |
| 62868 F | GTTAAATAACAGCCTGCTCCTCA |
| 62868 R | GCAGTTAGTAATTGTCAAGGCGA |
| 6820 F | GCAATACCAATCCAAACCCAT |
| 6820 R | CCTGGCTACAAAACGGACAAT |

Supplementary Table S4: Completeness evaluation of *S. asper* transcriptome using BUSCO.

| Reference | Complete BUSCOs | | Fragmented | Missing | Total |
| --- | --- | --- | --- | --- | --- |
|  | Single-copy | Duplicated | BUSCOs | BUSCOs | detected (%) |
| Eukaryotes | 215 (70.95%) | 65 (21.45%) | 17 (5.62%) | 6 (1.98%) | 98.02 |
| Viridiplantae | 307 (71.39%) | 36 (8.37%) | 54 (12.56%) | 33 (7.62%) | 93.32 |

Supplementary Table S5: Taxonomic assignment of assembled unigenes across the tree of life.

| Total | Unigenes | Percent (%) |
| --- | --- | --- |
| Archaea | 3 | 0.018 |
| Bacteria | 46 | 0.273 |
| Eukaryota | 16807 | 99.579 |
| Viruses | 22 | 0.130 |

| Eukaryota | Unigenes | Percent (%) |
| --- | --- | --- |
| Animals | 200 | 1.190 |
| Fungi | 91 | 0.541 |
| Plants | 16475 | 98.025 |
| Others | 41 | 0.244 |

Supplementary Table S6: Relative distribution of the various lineages among specifically expressed genes and DEGs (see Results).

| Origin | Specific | Regulated | Total | Percent assembly (%) |
| --- | --- | --- | --- | --- |
| Animals | 1 | 8 | 9 | 4.50 |
| Bacteria | 5 | 0 | 5 | 10.87 |
| Fungi | 37 | 15 | 52 | 57.14 |
| Others | 1 | 0 | 1 | 2.44 |
| Plants | 10 | 50 | 60 | 0.36 |
| Unknown | 16 | 13 | 29 | 0.14 |
| Viruses | 6 | 6 | 12 | 54.55 |
